# Supplementary material for: A functional theory of bistable perception based on dynamical circular inference
Source: PLoS Comput Biol. 2020 Dec 14;16(12):e1008480. doi: 10.1371/journal.pcbi.1008480 (PMC7769606; doi:10.1371/journal.pcbi.1008480)
Supplement: S1 Text — (DOCX) [file pcbi.1008480.s001.docx]

**S1 Text: Mathematical derivations**

**From belief propagation to the discrete model**

Belief propagation (BP; or Sum-Product algorithm) is a general and efficient algorithm that performs inference in directed acyclic graphs (DAG) (see the figure below **(A)**; [1]). In the most general case, the DAG has to be transformed into a factor graph (FG), which elucidates the factorization properties of the underlying joint distribution (**B**). A FG contains 2 types of nodes: variable nodes represented by lower-case indices (e.g., $i,j$) and factor nodes represented by upper-case indices (e.g., $I,J$), connected with each other through edges. All the variable nodes directly connected to a factor node (e.g., $I$) appear in the corresponding factor ($f_{I}$) and are represented by $x_{N_{I}}$.

BP works by propagating local messages between neighbouring nodes. There are 2 types of messages: messages sent from variables to factors and messages sent from factors to variables. Those messages can be computed recursively by the following equations (discrete variables):

$$\mu_{j\to I}\left( x_{j} \right)=\prod_{J\epsilon N_{j}\backslash\{I\}} \mu_{J\to j}\left( x_{j} \right) (S1)$$

$$\mu_{I\to i}\left( x_{i} \right)=\sum_{x_{N_{I}}\{i\}} f_{I}\left( x_{N_{I}} \right)\prod_{j\in N_{I}\backslash\{i\}} \mu_{j\to I} (S2)$$

$N_{j}$ is the set of all factors directly connected to variable $j$. Given that the FG is derived from a DAG, the factor $f_{I}(x_{N_{I}})$ is a conditional probability and represents the strength of the connection between the variables in $x_{N_{I}}$. Eq. $(S1)$ simply means that the message from a variable *j* to a factor $I$ is the product of all the messages arriving at *j*, except for the message sent from this particular factor $I$. Similarly, a message sent from a factor $I$ to a variable $i$ is the product of all the messages arriving at $I$, except for the message sent from $i$, weighed by the factor $f_{I}$ and marginalized over all the other variables in $x_{N_{I}}$ ($(S2)$). Once all messages have been propagated in both directions (one pass is sufficient for convergence to the correct posterior, when there are no loops), posterior probabilities can be computed as follows:

$$b_{i}\left( x_{i} \right)=\frac{1}{Z}\prod_{I\in N_{i}} \mu_{I\to i}(x_{i}) (S3)$$

where $Z$ is the normalization constant. Consequently, posteriors can be calculated as the products of all the messages arriving at each node.

In a previous paper, Jardri and Denève drew an analogy between BP and neural processing in recurrent, hierarchical networks (**C;** [3]). They showed that, when considering pairwise graphs and binary variables and taking the log-ratios, factors can be omitted and BP recursive equations can be rewritten as follow:

$$B_{i}=\sum_{j\in N_{i}} M_{j\to i} (S4)$$

$$M_{j\to i}=F\left( B_{j}-M_{i\to j},w_{ji}^{1},w_{ji}^{0} \right) (S5)$$

In $(S4)$ and $(S5)$, $M_{j\to i}=\log(\frac{\mu(x_{i}=1)}{\mu(x_{i}=0)})$ and $B_{i}=\log(\frac{b_{i}(x_{i}=1)}{b_{i}(x_{i}=0)})$ (log-ratio of messages and beliefs). $F()$ on the other hand corresponds to a sigmoid function, defined in the following way:

$$F\left( B,w^{1},w^{0} \right)=\log\left( \frac{w^{1}e^{B}+w^{0}}{\left( 1-w^{1} \right)e^{B}+\left( 1-w^{0} \right)} \right) (S6)$$

$w_{ji}^{1}$ and $w_{ji}^{0}$ correspond to the strength of the $(j\to i)$ connection and are defined as the following conditional probabilities:

$$w_{ji}^{1}=P\left( x_{i}=1 | x_{j}=1 \right), w_{ji}^{0}=P\left( x_{i}=1 | x_{j}=0 \right) (S7)$$

In equation $(S5)$, the message is defined as a function of the belief of the variable that sends the message, minus the message sent in the opposite direction. This correction (similar to the correction in the initial equations $(S1),(S2)$) is crucial, because it prevents the formation of circularity, i.e. reverberation of messages which are counted many times. Failure of the corrective mechanism (hypothesized to be implemented by inhibition) to subtract efficiently all the redundant information (e.g. due to E/I imbalance in favour of excitation) leads to circular inference (CI) (**D**), in which case equation $(S5)$ is written as follows:

$$M_{j\to i}=F\left( B_{j}-a_{S}^{'}M_{i\to j},w_{ji}^{1},w_{ji}^{0} \right),if i is below j (S9)$$

$$M_{j\to i}=F\left( B_{j}-a_{P}^{'}M_{i\to j},w_{ji}^{1},w_{ji}^{0} \right),if i is above j (S10)$$

Parameters $a_{S}^{'}$ and $a_{P}^{'}$ quantify the strength of the loops (ascending and descending respectively) per layer, in other words what part of each message gets reverberated within a single connection and take values between 0 and 1 (1 corresponds to exact inference, i.e. E/I balance).

Although the outcome of BP (and of CI) can be calculated recursively via eq. $(S4),(S5)$ ($(S4),(S9),(S10)$ for CI), the presence of the non-linear $F$ term makes the derivation of general and handy closed form solutions a difficult task. For this reason, a simplification of the BP scheme was recently suggested, that keeps all the essential features of CI while presenting it in a more operational form (**E;** [2]). In particular, the hierarchy is reduced to a 3-node graph, comprising the prior information, the sensory information and the variable whose posterior needs to be calculated. Because the total effect of the loops depends on the number of possible reverberations (i.e. the number of layers in the hierarchy), parameters $a_{S}^{'}$ and $a_{P}^{'}$ where replaced by $a_{S}$ and $a_{P}$, which can take any value above 0 (with 0 corresponding to exact inference; values below 0 are also possible and signify an increased strength of inhibition) and quantify the overall amplification of information in the whole original hierarchy. This simplified CI model can be written as follows:

$$L=F\left( L_{S}+R_{S}+R_{P},w_{S},1-w_{S} \right)+F\left( L_{P}+R_{S}+R_{P},w_{P},1-w_{P} \right) (S11)$$

where$L$ is the log-odds, $L_{S}$ is the log-likelihood ratio, $L_{P}$ is the log-prior ratio, $w_{S}$ is the feedforward weight, $w_{P}$ is the feedback weight and $R_{S},R_{P}$ are the reverberated terms, computed as follows:

$R_{S}=F\left( a_{S}L_{S},w_{S},1-w_{S} \right) (S12)$

$$R_{P}=F\left( a_{P}L_{P},w_{P},1-w_{P} \right) (S13)$$

For Gaussian noise, the log-likelihood ratio can be written in the following way:

$$L_{S}=2\frac{\mu_{int}}{\sigma_{\iota nt}^{2}}S_{t} =wS_{t} (S14)$$

where: $S_{t}\sim N(\mu_{noise},\sigma_{noise}^{2})$

and ($\mu_{int}, \sigma_{int}, \mu_{noise}, \sigma_{noise}$) are parameters of the internal / noise model (**Main Text**).

In the discrete case, that corresponds to a random walk around $\mu_{noise}$.

In eq. $(S11)$ the first term represents the total bottom up information and the second term the total top down information. That comprises the original information (likelihood and prior respectively) along with the reverberated terms, that means the likelihood corrupting the prior and vice versa. Furthermore, an additional term is considered inside each component (a reverberated likelihood for the likelihood term and a reverberated prior for the prior term) that renders the 2 streams practically indistinguishable.

Both implementations of CI make the same assumptions and generate the same qualitative predictions. More particularly, they both hypothesize that redundant information is not fully removed from the propagated messages, leading to amplification of sensory evidences and/or priors [3,4]. Additionally, bottom-up information is corrupted by top-down information and vice versa, creating aberrant correlations between sensory evidence and priors ([2,4,5]).

The model presented in this paper is similar to the model described by eq. $(S11)$ but it comprises dynamics too, resulting in the following equation (**F**):

$$L_{t+1}=F\left( L_{S}+R_{S}+R_{P},w_{S},1-w_{S} \right)+F\left( L_{t}+R_{S}+R_{P},1-r_{off},r_{on} \right) (S15)$$

In $(S15)$ we assume$dt=1$ (discrete model). $L_{t}$ is the log-posterior ratio of variable $X$ (3D interpretation) at time t, which becomes the prior in the next time step. $r_{on},r_{off}$ are the transition rates (see **Main Text**), which for simplicity and without loss of generality have been taken equal to the feedback weights ($r_{on}=w_{P}^{0},r_{off}=1-w_{P}^{1};$ in $\left( S13 \right)$ we assumed $w_{P}^{1}=w_{P},w_{P}^{0}=1-w_{P}$).

Note that the 2 rates are not necessarily equal to each other. In that case, $F(0,1-r_{off},r_{on})\neq0$. In order to avoid reverberation of information in the absence of descending loops ($a_{P}=0$), we defined $R_{P}$ as follows:

$$R_{P}=F\left( a_{P}L_{t},1-r_{off},r_{on} \right)-F\left( 0,1-r_{off},r_{on} \right) (S16)$$

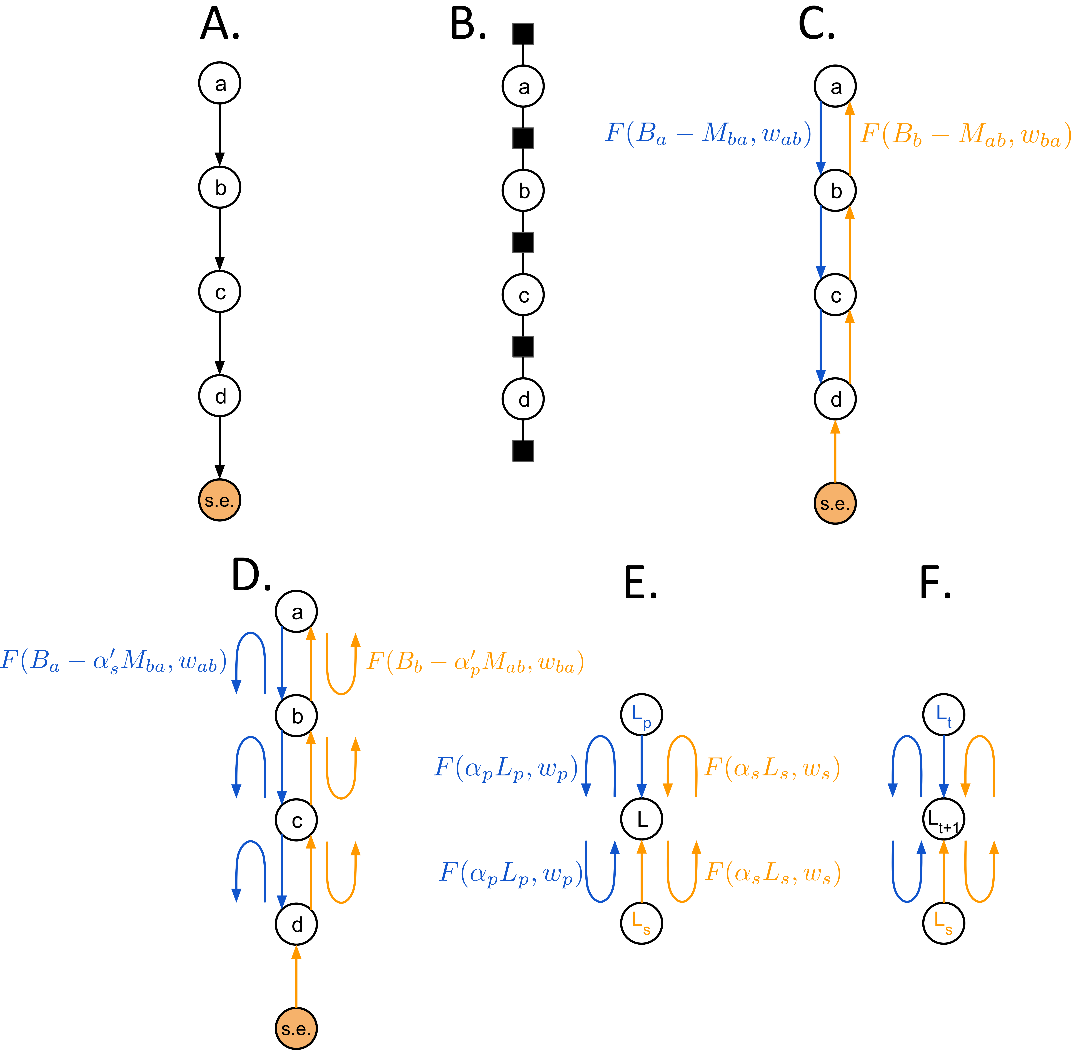


***BP and discrete model. (A.):*** *The generative model as Bayesian network.* ***(B.):*** *The factor graph.* ***(C.):*** *Belief propagation.* ***(D.):*** *Circular inference.* ***(E.):*** *A simplified model for circular inference* [2]*.* ***(F.):*** *The discrete dynamical model.*

**Mathematical derivation of the continuous model**

We now consider infinitesimally small time-steps ($dt\to0$) and the corresponding sensory evidence ${dS}_{t}$. We assume $r_{on},r_{off}$ and $a_{P}$ to be proportional to $dt$. Then equation $(S15)$ becomes (after using $(S12),(S14)$ and $(S16)$):

$$L_{t+dt}=$$

$$F\left( w{dS}_{t}+F\left( a_{S}w{dS}_{t},w_{S},1-w_{S} \right)+F\left( a_{P}dtL_{t},1-r_{off}dt,r_{on}dt \right) -F\left( 0,1-r_{off}dt,r_{on}dt \right),w_{S},1-w_{S} \right)+$$

$$+F\left( L_{t}+F\left( a_{S}w{dS}_{t},w_{S},1-w_{S} \right)+F\left( a_{P}dtL_{t},1-r_{off}dt,r_{on}dt \right) -F\left( 0,1-r_{off}dt,r_{on}dt \right),1-r_{offdt},r_{on}dt \right)=$$

$$=F_{S}+F_{P} (S17)$$

We linearize equation $(S17)$ using the Taylor expansion of each term and keeping only the first order terms.

The following general equalities hold (see **Derivations** below):

$$F\left( x,w,1-w \right)=\left( 2w-1 \right)x+O\left( x^{2} \right), for x\to0 (S18)$$

$$F\left( x,1-r_{off}dt,r_{on}dt \right)=x+dt\left( r_{on}\left( 1+e^{-x} \right)-r_{off}\left( 1+e^{x} \right) \right)+O\left( x^{2} \right) (S19)$$

$$\log\left( 1+x \right)=x+O\left( x^{2} \right), for x\to0 (S20)$$

Using $(S18)$-$(S20)$ we get:

$$F\left( a_{S}w{dS}_{t},w_{S},1-w_{S} \right)=\left( 2w_{S}-1 \right)a_{S}wdS_{t}+O\left( {dt}^{2} \right) (S21)$$

$$F\left( a_{P}dtL_{t},1-r_{off}dt,r_{on}dt \right)=a_{P}dtL_{t}+dt\left( r_{on}\left( 1+e^{-a_{P}dtL_{t}} \right)-r_{off}\left( 1+e^{a_{P}dtL_{t}} \right) \right)+O\left( {dt}^{2} \right)(S22)$$

$$F\left( 0,1-r_{off}dt,r_{on}dt \right)=2dt\left( r_{on}-r_{off} \right)+O\left( {dt}^{2} \right) (S23)$$

From $(S22)$, using $(S20)$, we get:

$F\left( a_{P}dtL_{t},1-r_{off}dt,r_{on}dt \right)=a_{P}dtL_{t}+2dt\left( r_{on}-r_{off} \right)+O\left( {dt}^{2} \right) (S24)$

Using $(S18)$-$(S24)$, we conclude:

$$F_{S}=w\left( 2w_{S}-1 \right)\left( 1+a_{S}\left( 2w_{S}-1 \right) \right)dS_{t}+a_{P}dt\left( 2w_{S}-1 \right)L_{t}+ O\left( {dt}^{2} \right) (S25)$$

$$F_{P}=L_{t}+a_{S}w\left( 2w_{S}-1 \right){dS}_{t}+a_{P}dtL_{t}+dt\left( r_{on}\left( 1+e^{-L_{t}} \right)-r_{off}\left( 1+e^{L_{t}} \right) \right)+ O\left( {dt}^{2} \right) (S26)$$

Using $(S25)$ and $(S26)$, $(S17)$ becomes:

$$L_{t+dt}=L_{t}+2w_{S}a_{P}{dtL}_{t}+dt\left( r_{on}\left( 1+e^{-L_{t}} \right)-r_{off}\left( 1+e^{L_{t}} \right) \right) +w\left( 2w_{S}-1 \right)\left( 1+2w_{S}a_{S} \right)dS_{t}+O\left( {dt}^{2} \right) (S27)$$

Then:

$$\frac{dL}{dt}=\frac{L_{t+dt}-L_{t}}{dt}=2w_{S}a_{P}L+(r_{on}e^{-L}-r_{off}e^{L})+(r_{on}-r_{off})+w_{int}\left( 2w_{S}-1 \right)(1+2w_{S}a_{S})S$$

which is the equation of the **Main text** and $S$ is a Gaussian stochastic process.

**Derivation of** $(S18)$

Using the definition of $F()$ (eq. $S(6)$) and the Taylor expansion: $e^{x}=1+x+O(x^{2})$, we get:

$$F(x,w,1-w)=\log(w(1+x+O(x^{2}))+1-w)-\log((1-w)(1+x+O(x^{2}))+w)$$

$$=log(1+wx+O(x^{2}))-\log(1+x-wx+O(x^{2}))$$

Using $(S20)$, we get $(S18)$

**Derivation of eq.**$(S19)$

Using the definition of $F()$ (eq. $S(6)$):

$$F(x,1-r_{off}dt,r_{on}dt)=x+\log(1+dt(-r_{off}+r_{on}e^{-x}))-\log(1+dt(r_{off}e^{x}-r_{on}))$$

Using $(S20)$, we get $(S19)$

**References**

1. Bishop C. Pattern Recognition and Machine Learning. Springer; 2006.

2. Jardri R, Duverne S, Litvinova AS, Denève S. Experimental evidence for circular inference in schizophrenia. Nat Commun. 2017;8: 14218. doi:10.1038/ncomms14218

3. Jardri R, Denève S. Circular inferences in schizophrenia. Brain. 2013;136: 3227–41. doi:10.1093/brain/awt257

4. Leptourgos P, Denève S, Jardri R. Can circular inference relate the neuropathological and behavioral aspects of schizophrenia? Curr Opin Neurobiol. 2017;46: 154–161. doi:10.1016/j.conb.2017.08.012

5. Leptourgos P, Notredame CE, Eck M, Jardri R, Denève S. Circular inference in bistable perception. J Vis. 2020;20: 12. doi:10.1167/jov.20.4.12
