# Supplementary material for: A functional theory of bistable perception based on dynamical circular inference
Source: PLoS Comput Biol. 2020 Dec 14;16(12):e1008480. doi: 10.1371/journal.pcbi.1008480 (PMC7769606; doi:10.1371/journal.pcbi.1008480)
Supplement: S2 Text — (DOCX) [file pcbi.1008480.s002.docx]

**S2 Text: Bifurcation analysis**

***Pitchfork bifurcation***

We now consider the case when there is no stimulation.

If $r_{on}=r_{off}=r$, we get:

$$f_{L}=\frac{df}{dL}=2w_{S}a_{P}-r\left( e^{-L}+e^{L} \right) (S28)$$

A pitchfork bifurcation occurs at the maximum value of $a_{P}$, for which $f_{L}<0,\forall L$ (and consequently $f$ is monotonically descending $\to$ there is one stable fixed point) (**Fig 4A and 4B**)

For $a_{P}<0$ $\to$ $f_{L}<0,\forall L$

For $a_{P}\geq0$, the first term in $(S28)$ is always non-negative, while the second term is always negative. Then we need: $2w_{S}a_{P}<r(e^{-L}+e^{L}),\forall L$

We know that:

$$min\left( r\left( e^{-L}+e^{L} \right) \right)=2r (S29)$$

Then:

if $2w_{S}a_{P}<2r\leftrightarrow a_{P}<\frac{r}{w_{S}}$, $f_{L}<0,\forall L$

Consequently, a pitchfork bifurcation occurs when:

$$a_{P}^{Pf}=\frac{r}{w_{S}}$$

For $a_{P}<\frac{r}{w_{S}}$, there is one stable fixed point at $L=0$. For $a_{P}>\frac{r}{w_{S}}$, there is an unstable fixed point at $L=0$ and two stable fixed points given by the following equation:

$f(L_{fp})=$ $2w_{S}a_{P}L_{fp}+r\left( e^{-L_{fp}}-e^{L_{fp}} \right)=0 (S30)$

The different cases are presented in the figure below (see also **Fig 3C**).


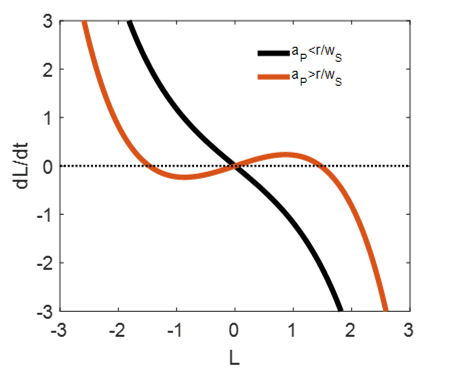


***The Pitchfork Bifurcation***

***Saddle-Node bifurcation***

If $r_{on}\neq r_{off}$, we get:

$$f_{L}=\frac{df}{dL}=2w_{S}a_{P}-\left( r_{on}e^{-L}+r_{off}e^{L} \right) (S31)$$

If $a_{P}=0$, there is one stable fixed point at $L=\log(\frac{r_{on}}{r_{off}})$ (**MainText;** see also the figure below (black)).

If we extend the argument outlined for the case of the pitchfork bifurcation, we find that function $f$ stops being monotonically descending when:

$$a_{P}=\frac{A}{w_{S}} (S32)$$

where: $A=r_{on}\sqrt{\frac{r_{off}}{r_{on}}}+r_{off}\sqrt{\frac{r_{on}}{r_{off}}} (S33)$

Because of the asymmetry introduced by the 2 rates, that is not a bifurcation point (see the figure below (green)). Instead, a Saddle-Node bifurcation occurs when one of the two local extrema crosses x-axis (**Fig 4C and 4D**). For the local extrema the following holds:

$$f_{L}=0\to L_{1,2}=\log\left( \frac{w_{S}a_{P}\mp\sqrt{w_{S}^{2}a_{P}^{2}-r_{on}r_{off}}}{r_{off}} \right) (S34)$$

Then, we can calculate the value (of $a_{P},r_{on}$ or $r_{off}$) at which SN bifurcation occurs simply by taking: $f(L_{1,2})=0$

All the fixed points can be calculated from the following equation (see the figure below (magenta)):

$f\left( L_{fp} \right)=2w_{S}a_{P}L_{fp}+r_{on}e^{-L_{fp}}-r_{off}e^{L_{fp}}+\left( r_{on}-r_{off} \right)=0 (S35)$


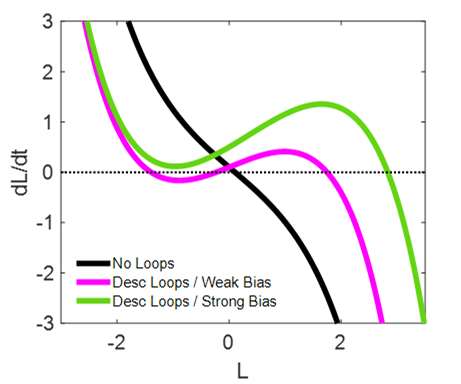


***The*** ***Saddle-Node bifurcation.*** *The green curve corresponds to an extremely strong implicit bias (the system gets stuck to interpretation 1)*
