# Supplementary material for: A functional theory of bistable perception based on dynamical circular inference
Source: PLoS Comput Biol. 2020 Dec 14;16(12):e1008480. doi: 10.1371/journal.pcbi.1008480 (PMC7769606; doi:10.1371/journal.pcbi.1008480)
Supplement: S3 Text — (DOCX) [file pcbi.1008480.s003.docx]

**S3 Text: Phenomenology of bistable perception**

From a phenomenological point of view, bistability is a unique experience. Prolonged viewing of an ambiguous stimulus generates unstable percepts that switch between two configurations, despite the stimulus being exactly the same. Perception in that case seems to be dissociated from stimulation. Additionally, despite the ambiguous and unreliable nature of the stimulus, each interpretation usually persists for many seconds before switching back, and most of the time, it is perceived with high levels of confidence. This fascinating phenomenon leads to several unresolved questions. For instance, why would a perceptual system choose to favour one interpretation instead of the other or why choose one interpretation instead of perceiving both at the same time? In addition to that, why would that system change its mind? Finally, how does this system generate strong beliefs in the absence of strong evidence? Following Hohwy and colleagues, we call the first question the “*selection problem*” and the second question the “*alternation problem*” [1]. Moreover, we call the third question the “*confidence problem*”.

In agreement with previous studies, we argue that “selection” is a simple consequence of the brain’s function: to make “perceptual” predictions under uncertainty [2,3]. According to this hypothesis, the brain chooses the cause that best explains its sensory evidence. If at a given moment the sensory evidence, combined with any available prior knowledge (e.g., SFA preference) supports more strongly a certain interpretation, this interpretation will be chosenand perceived by the brain.

Furthermore, apart from the sensory evidence and the priors, additional information (e.g., epistemological truths) might have to be considered when solving ill-posed problems, such as the problem of 3D perception. For example, common sense dictates that two different objects cannot occupy the same part of the visual space [1]. We postulate that such a hyperprior renders the 2 interpretations mutually exclusive, and consequently impossible to be perceived at the same time.

We further argue that the “*alternation problem*” is a consequence of another property of the brain: evidence accumulation. In the model, this is captured by the Markovian statistics and it is regulated by two transition rates. More particularly, the new noisy evidence is integrated into the past accumulated evidence and pushes the belief away from its stable state. Every time the belief crosses the threshold, a switch occurs.

Although the “*selection*” and the “*alternation*” problems can be solved rather easily by referring to general functions of the brain, the “*confidence*” problem seems more difficult to deal with. Despite being unstable, percepts usually persist many seconds before switching, while they are also held with high levels of confidence. On the contrary, a system doing exact inference in the presence of some environmental volatility (i.e., a system without loops and with non-zero transition rates) would switch very often, since the belief would have the tendency to hover around the prior (**Fig 2B**). This problem could be resolved by considering a perfect integrator ($r_{on}=r_{off}=0$). However, such a system is not well-suited for unstable environments. In addition to that, even a perfect integrator is not able to generate strong beliefs in the absence of strong and reliable data, as for example when facing bistable stimuli. Crucially, both persistence and high confidence would be expected from a system that over-counts its accumulated evidence (prior knowledge), i.e. a system with descending loops. As shown in **Fig 2A and 2C**, even weak descending loops give rise to extremely strong beliefs, generating a system that is very confident even in the face of noisy and ambiguous evidence. At the same time, descending loops increase the persistence of the percepts, by amplifying the stabilizing effect of past information. This dual effect of the descending loops is due to their ability to change the dynamics of the system - they transform it into a bistable attractor.

**References**

1. Hohwy J, Roepstorff A, Friston K. Predictive coding explains binocular rivalry: An epistemological review. Cognition. 2008;108: 687–701. doi:10.1016/j.cognition.2008.05.010

2. Brascamp J, Sterzer P, Blake R, Knapen T. Multistable Perception and the Role of the Frontoparietal Cortex in Perceptual Inference. Annu Rev Psychol. 2018;69: 1–27.

3. Weilnhammer V, Stuke H, Hesselmann G, Sterzer P, Schmack K. A predictive coding account of bistable perception - a model-based fMRI study. PLoS Comput Biol. 2017;13: 1–21. doi:10.1371/journal.pcbi.1005536
