# Supplementary material for: A functional theory of bistable perception based on dynamical circular inference
Source: PLoS Comput Biol. 2020 Dec 14;16(12):e1008480. doi: 10.1371/journal.pcbi.1008480 (PMC7769606; doi:10.1371/journal.pcbi.1008480)
Supplement: S4 Text — (DOCX) [file pcbi.1008480.s004.docx]

**S4 Text: Parameter Recovery**

Without loss of generality, our dynamical circular inference model can be reduced to 4 free parameters: the two transition rates $r_{on}$ and $r_{off}$, the auto-amplification $a$ and the overall gain of the sensory inputs $w_{int}^{*}$. Here we explored whether we can accurately recover those parameters from simulated data. We chose to simulate an intermittent presentation task (with various OFF-Durations), but a similar method can be used to fit the model to different types of data, e.g. continuous presentation tasks, tasks with confidence reports etc.

The parameter recovery procedure is the following: We generated “fake” intermittent presentation data using random parameters from a certain range ($w_{int}^{*}$: [0.35 , 100]; $r_{on}$ and $r_{off}$: [0 , 2.5]; $a$: [0 , 10]). We fitted the dynamical circular inference model to the “fake” data by minimizing the mean-squared error between the survival probability (for both possible percepts and for several off durations) of the “fake” data and that of the generated data. The optimization algorithm used is Matlab’s *patternsearch* function.

For intermittent presentation, parameters can be recovered accurately, provided that the ($r_{on}+r_{off}$) is known. ($r_{on}+r_{off}$) is a measure of the volatility and one can assume that it corresponds to a typical visual integration time constant, about 100-200 ms (here volatility was fixed to 200 ms). The first figure below illustrates the quality of the parameter recovery (for all 4 parameters, the Pearson’s correlation coefficient between “real” and estimated parameters was greater than 0.9), while the second figure illustrates a strong overlap between “real” (thick solid lines) and estimated (dotted lines) survival probabilities.

For unknown volatility, the sensory gain $w_{int}^{*}$ and the amount of loops $a$ can be underestimated (respectively overestimated) if the volatility ($r_{on}+r_{off}$) is overrestimated (respectively underestimated) (see the third figure below). It is worth highlighting though that even when there are no constraints in the parameters, the correlation between “real” and estimated values is strong, allowing for group comparisons (e.g. healthy controls against schizophrenia patients). Alternatively, one could aim at observing how quickly the confidence drops when it is initially very high. Indeed, a Taylor expansion of the dynamical equation gives :

$$\frac{dL}{dt}=aL+r_{on}e^{-L}-r_{off}e^{L}+r_{on}-r_{off}+w_{int}^{*}S$$

$$\approx2\left( r_{on}-r_{off} \right)+\left[ a-\left( r_{on}+r_{off} \right) \right]L+\frac{r_{on}-r_{off}}{2}L^{2}-\frac{r_{on}+r_{off}}{6}L^{3}\ldots+w_{int}^{*}S (S36)$$

meaning that the bias ($r_{on}-r_{off}$) and the difference $a-\left( r_{on}+r_{off} \right)$ between the amount of loops and the volatility determine the evolution of the confidence around the energy barrier between the 2 attractor states. In order to observe the effects of the higher-order terms in L, allowing to determine the volatility (term of order 3 in L) and thus all parameters, one could for instance sometimes replace the ambiguous Necker cubes by unambiguous ones. In that case, an increase of the OFF-Duration (following the unambiguous cube) would decrease the stability of the percept until reaching the same level as for the ambiguous cube. How quickly that happens is determined by the volatility.


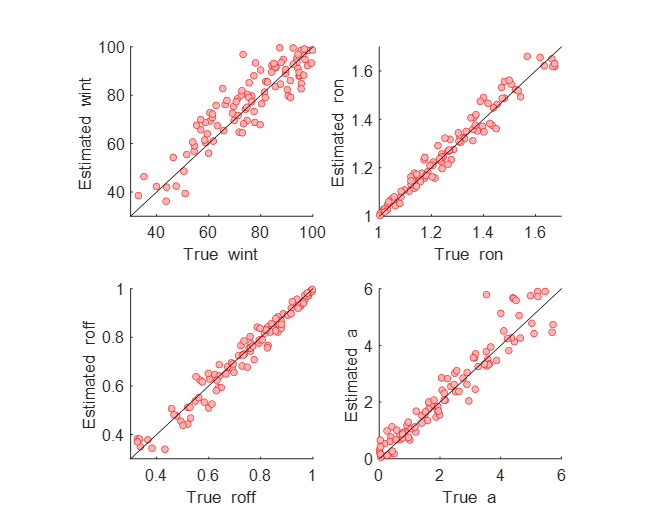


***Parameter recovery – known volatility (***$\boldsymbol{r}_{\boldsymbol{on}}\boldsymbol{+}\boldsymbol{r}_{\boldsymbol{off}}$***).*** *The correlation between true and estimated parameters is strong for all 4 parameters:* $r_{w_{S}}=0.90$*,*$r_{r_{on}}=0.98$*,*$r_{r_{off}}=0.98$*,*$r_{a}=0.96$*; p<<0.001.*

**
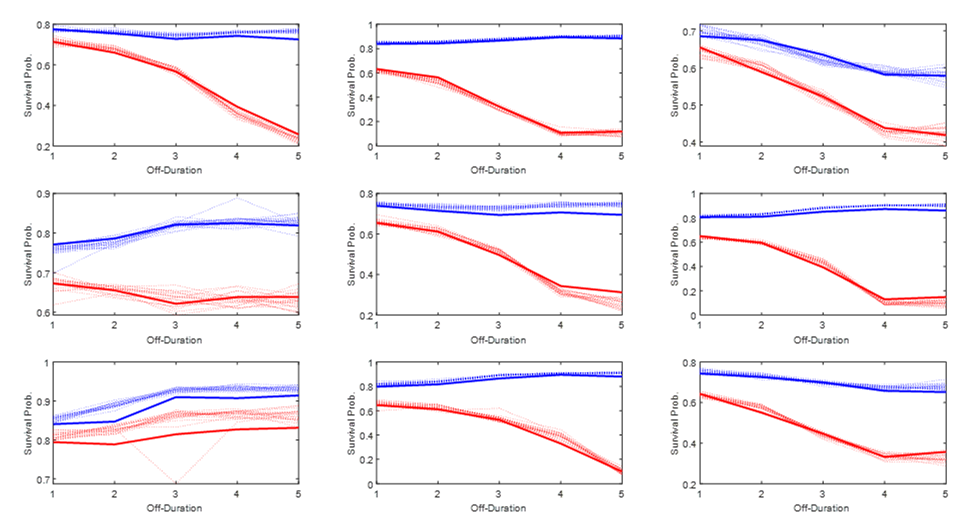
**

***Real and estimated survival probabilities - Parameter recovery for known volatility.*** *Thick solid lines: “real” survival probabilities; dotted lines: estimated survival probabilities; blue / red color: SFA / SFB interpretation. Each panel corresponds to a random set of parameter values (a data point from the figure above). For the estimated survival probabilities, simulations were repeated 20 times. There is a good overlap between “real” and estimated survival probabilities.*

**
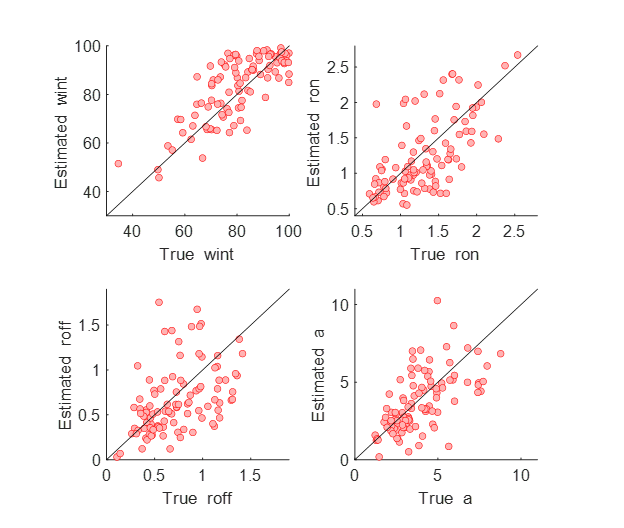
**

***Parameter recovery – unknown volatility.*** *Parameter recovery was less accurate but the correlation between true and estimated parameters is still strong for all 4 parameters:* $r_{w_{S}}=0.77$*,*$r_{r_{on}}=0.62$*,*$r_{r_{off}}=0.45$*,*$r_{a}=0.61$*; p<<0.001.*
