# Supplementary material for: A functional theory of bistable perception based on dynamical circular inference
Source: PLoS Comput Biol. 2020 Dec 14;16(12):e1008480. doi: 10.1371/journal.pcbi.1008480 (PMC7769606; doi:10.1371/journal.pcbi.1008480)
Supplement: Attachment — Submitted filename: PlosComp_biol_L.pdf [file pcbi.1008480.s005.pdf]

The authors present a new computational approach toward bistable perception. Based on the established algorithm of circular inference, they deduce minimal conditions under which bistable perception can occur in this framework. With this, the authors place bistable perception in the general context of perceptual inference. Lastly, they relate their model to alternations in perceptual inference related with psychotic symptoms.

Next to analytical methods, the authors performed simulation analyses and compared the model predictions to a number of empirical characteristics of bistable perception (Levelt's laws and the stabilization of bistable perception by intermittent presentation of ambiguous stimuli).

I think that the circular-inference approach to bistable perception outlined in the manuscript is highly relevant for two reasons: Firstly, it deduces the perceptual dynamics of bistable perception from general considerations of perceptual inference. To my mind, such a functional take on bistability is important, since it may help translate perceptual phenomena observed largely in a laboratory context (eg, the frequent perceptual transitions experienced during perceptions) to the characteristics of perception in real-world scenarios. Secondly, by inverting the circular inference model based on behavior, researchers may be able to quantify the relative contribution sensory evidence and prior knowledge on perceptual inference. This may proffer new opportunities in the study of alterations in perceptual inference (eg, positive psychotic symptoms in schizophrenia).

Although I am very sympathetic toward this work, I have several concerns and wishes for clarification:

General Comments

First, broadly, the authors' claim that this model can be fitted to behavioral data (and thus be useful to study perceptual function in health and disease) does not seem to be backed up empirically in the manuscript. Personally, I would think that the authors could strengthen their circular-inference model of bistable perception significantly if they could show that the latent variables of the model (eg, weights for *ascending/descending loops*, feed-forward weight *ws*, the response variable *theta,* rates of change r_{on}/r_{off}) can be reliably estimated from data. To my mind, this would mean that, when simulating behavioral responses for a set of latent variables, such latent variables could be recovered from the simulated data.

Second, I have some specific wishes for further clarification regarding the methods and analysis, which I outline in detail below. Lastly, I have a few recommendation on how to improve the connection of the authors' findings to the existing literature. Specific comments are below.

Major comments:

1. I did not fully understand the role of the decision threshold theta. To my understanding, in this circular-inference (CI) model, setting theta to a sufficiently high value should be necessary to maintain stable perceptual states (view from above; view from below in the example of the Necker Cube). For low values of theta, I could imagine that spontaneous fluctuation in L should lead to frequent switches between dominant perceptual states. Yet, in the method section, the authors note that:

"(…) in the case of continuous presentation, it is necessary to set θ to a sufficiently high positive value to obtain robust perceptual switches. If θ = 0, the percept would switch multiple times

(just because of the noisy input causing small random fluctuations in L) around the time of perceptual transitions."

> Specifically, I did not understand why the stabilizing effect of a high decision threshold stabilized perception only around the time of perceptual transitions,

2. On a more general view, I would find it helpful to see an illustration of the effects of theta on the model's predictions.

"In our model, a nonzero decision threshold precludes percepts with very short durations. As a result, the distribution resembles a gamma or log-normal distribution. The rising phase and peak of this distribution depend on the decision threshold, but the tail of the distribution does not and is imposed by the mathematical properties of bistable dynamics driven by noise."

> Did I understand correctly that, in the presence of descending loops, it is only due to the decision threshold that simulated phase durations are distributed in a gamma/log-normal distribution? Is the location of the peak of the distribution uniquely determined by the value of the decision threshold? Is there any relation between the energy landscapes shown in Figure 3 and the theta parameter? Moreover, is the minimum value of the decision threshold that is necessary to induce stable perceptual inference correlated with the standard deviation of sensory evidence / the likelihood function?

3. In a related point, I would need additional clarifications on how the role of theta relates to the function of descending loops:

"Note that large values of θ can lead to a distribution of phase duration similar to the system of descending loops. However, while the distribution of phase duration cannot be considered proof of the presence of circular inference, the resulting confidence is often below the decision thresholds. This may preclude the emergence of strong and stable percepts in the absence of descending loops."

> Here, the authors introduce the concept of "confidence". If I understood it correctly, high-confidence perceptual states only emerge in the presence of descending loops. I would find it helpful if the authors could contextualize this to the existing literature on bistable perception:
> A number of studies has devoted a lot of attention of mixed percepts during bistability (eg., Knapen 2011). Do the authors assume that such mixed percepts (low-confidence/high-uncertainty perceptual states) arise at the time of state transitions between the energy wells in Figure 3c? How would the energy landscape look like for other types of bistable stimuli that show sudden transitions, such as discontinuous structure-from-motion stimuli (eg., Weilnhammer 2013)?

4. With regard to perceptual biases: From Figure 3d, should it be concluded that the CI model assumes a difference in confidence when there is a bias between perceptual alternatives? In the example of the Necker Cube, this would mean that the view-from-below is generally associated with reduced confidence. To my mind, this would be an important prediction of the CI model. Are the authors aware of any empirical evidence for this model prediction?

5. In the section on Levelt's 4th law, the authors investigate the effect on an increase in the strength of both interpretations on the alternation rate of a bistable stimulus. They

captured this increase in stimulus strength by increasing the variance of the noise distribution. This choice did not seem straightforward to me. Several alternatives would also seem plausible to me: Could an increase in stimulus strength be reflected by a decrease in variance of the noise distribution? Or by a modulation in variance of the likelihood distribution?

Minor comments

With regard to the **Abstract**, I would like to make a few suggestions that could render the content more accessible to the naïve reader:

1. While these points become clear after reading the manuscript, my personal impression was that they are difficult to understand on the basis of the abstract and general knowledge about bistable perception. Readers without a background in computational modelling of bistable perception might have a hard time understanding these points.

"We show that in the face of ambiguous sensory stimuli, circular inference can turn what should be a leaky integrator into a bistable attractor switching between two highly trusted interpretations. (…) Since it is related to the generic perceptual inference mechanism, this approach can be used to predict the tendency of individuals to form aberrant beliefs from their bistable perception behavior."

2. Maybe I have overlooked something, but while the main text contains a section of psychotic symptoms in schizophrenia patients, I could not find a discussion on cognitive functions in non-clinical populations.

"Overall, we suggest that feedforward/feedback information loops in hierarchical neural networks, a phenomenon that could lead psychotic symptoms when overly strong, could also underlie cognitive functions in nonclinical populations."

With regard to the **introduction**, I have a few additional comments:

3. If I understood correctly, the authors introduced perceptual inference during bistable perception as "suboptimal":

"In most cases, this task is performed very accurately, and the correct interpretation is found. Sometimes, perceptual systems fail to detect any meaningful interpretation (e.g., when sensory evidence is too degraded) or converge to the wrong interpretation. Finally, a third possibility occurs (mainly in lab conditions [3]) when ambiguity is high; the system detects more than one plausible interpretations but instead of committing to one interpretation, it switches every few seconds, a phenomenon known as *bistable perception* [4].

(….) Crucially, there is a discrepancy between the real input and the input assumed by the internal model. This, together with the loops, predicts the suboptimal inference at the heart of bistable perception (Figure 1; caption).

I was wondering whether the authors could add a little more detail as to why they view perceptual inference is suboptimal or incorrect. As they authors note throughout the

manuscript, truly ambiguous images (eg. the line drawings of a Necker cube, disparate monocular inputs in case of binocular rivalry) are very rare.
Could it also be that, because of the extremely low probability of a fully ambiguous real-word cause of sensory input, committing to one highly trusted stimulus interpretation is indeed adaptive/optimal? This thought also appears in the discussion ("Moderate descending loops could improve the system, allowing rapid and robust decisions even when evidence is not conclusive")
