# Supplementary material for: A functional theory of bistable perception based on dynamical circular inference
Source: PLoS Comput Biol. 2020 Dec 14;16(12):e1008480. doi: 10.1371/journal.pcbi.1008480 (PMC7769606; doi:10.1371/journal.pcbi.1008480)
Supplement: Attachment — Submitted filename: PlosComp_biol_II.pdf [file pcbi.1008480.s007.pdf]

I would like to thank the authors for their very complete revisions. For me, only minor points remain to be resolved (see below).

General Comments:

C1: First, broadly, the authors' claim that this model can be fitted to behavioral data (and thus be useful to study perceptual function in health and disease) does not seem to be backed up empirically in the manuscript. Personally, I would think that the authors could strengthen their circular-inference model of bistable perception significantly if they could show that the latent variables of the model (eg, weights for ascending/descending loops, feed-forward weight ws, the response variable theta, rates of change ron/roff) can be reliably estimated from data. To my mind, this would mean that, when simulating behavioral responses for a set of latent variables, such latent variables could be recovered from the simulated data.

R1: We would like to thank the reviewer for this excellent suggestion. We have now included a "Parameter Recovery" section in the Supplementary Material.

CC1: The section "Parameter Recovery" is a great addition to the paper. I would suggest adding a legend to Figure S5 (what to red and blue colors stand for?). I would also recommend adding p-values to the correlations shown in the Supplementary Figures S4 and S6.

C2: I did not fully understand the role of the decision threshold theta. To my understanding, in this circular-inference (CI) model, setting theta to a sufficiently high value should be necessary to maintain stable perceptual states (view from above; view from below in the example of the Necker Cube). For low values of theta, I could imagine that spontaneous fluctuation in L should lead to frequent switches between dominant perceptual states. Yet, in the method section, the authors note that:"(...) in the case of continuous presentation, it is necessary to set to a sufficiently high positive value to obtain robust perceptual switches. If = 0, the percept would switch multiple times (just because of the noisy input causing small random fluctuations in) around the time of perceptual transitions."
Specifically, I did not understand why the stabilizing effect of a high decision threshold stabilized perception only around the time of perceptual transitions.

R2: We thank the reviewer for their comments / questions about theta. They have casted some doubt on the importance of the theta decision criterion and after some reflection, we decided to remove it from the paper

and replace it with a simple “maximum-a-posteriori” (theta = 0). The reasons are threefold: First, it’s an ad-hoc addition to the dynamical Circular Inference (dCI) model described in the manuscript (i.e. it is not derived from first principles) and as such, it might be obscuring the take-home message of the paper; second, even in the presence of a non-zero theta, gamma-like histograms (the main reason for adding theta) are generated only in a subset of the parameter space (e.g. for large theta, small sensory gain and mild loops); third, it’s a decision criterion rarely described in the literature. As a result, our dCI model cannot (in its current form) give rise to gamma-like phase duration histograms. We now acknowledge that in the Main Text (Discussion) and describe possible solutions, including the filtering of the sensory input, an adaptation-like mechanism (time-dependent transition rates) and the theta decision criterion (we specify that all of them constitute ad-hoc extensions of the model).

CC2: Thanks a lot for the clarifications. To my mind, the fact that simulated phase durations are exponential is an important point. Given that it is explicitly mentioned in the discussion, I would therefore recommend making this point more visible in the paper, e.g. by showing the histogram in Figure S6 in the main text.
Specifically, for this visualization, I would recommend a smaller bin size. Is there a reason for showing normalized phase duration? How would the distribution look like for pooled dominance durations in seconds?

Despite the removal of the theta parameter, we would like to briefly address the reviewer’s comment. As they correctly pointed out, the non-zero decision threshold theta introduces a stabilizing factor to the perceptual system, by increasing its robustness against noise. When theta = 0 (MAP decision criterion), a switch occurs each time L (log-odds) crosses chance level (L = theta = 0). The closer L gets to 0, the higher the switching probability, since inputs can push L to the other side of the threshold more easily. When L is in the vicinity of the threshold, tiny fluctuations of the input can cause multiple switches in a very short period of time (“the percept would switch multiple times around the time of perceptual transitions”), resulting in a large number of extremely short (and meaningless) phases (and exponential distribution of phase durations). When we add a non-zero decision threshold (theta=a), switches occur as follows: when L=a is crossed from below or when L=-a is crossed from above.
As a result of this belief-dependent decision threshold, the perceptual system becomes more conservative with regard to switches: a switch occurs only when there is substantial evidence against the current hypothesis; e.g., if the dominant percept switches from SFB to SFA

(because L crossed the upper threshold a), it cannot switch back to SFB simply by crossing the same threshold in the opposite direction. Instead, the perceptual system must accumulate evidence in favor of SFB and reach the lower threshold. Therefore, short phases become rare and, under certain circumstances, this can also result in gamma distributions of phase durations.

CC2: Thanks for the clarifications.

C5: With regard to perceptual biases: From Figure 3d, should it be concluded that the CI model assumes a difference in confidence when there is a bias between perceptual alternatives? In the example of the Necker Cube, this would mean that the view-from-below is generally associated with reduced confidence. To my mind, this would be an important prediction of the CI model. Are the authors aware of any empirical evidence for this model prediction?

R5: We would like to thank the reviewer for this excellent suggestion. This is indeed a strong prediction of the dCI model: When there is an asymmetry, the weaker interpretation is associated with reduced confidence. That's because a bias affects the depth and the position of the wells (Fig. 3d). That being said, testing this prediction could be problematic for two reasons. First, because it is very hard to (reliably) measure confidence in a bistable perception experiment, without interfering with bistability. Second, because the magnitude of the effect might be small, especially compared to the effect of bias on stability (see Fig 2c). Consequently, we are not aware of any evidence supporting (rejecting) this prediction.

CC5: I do not see a general problem in obtaining confidence reports during bistability (e.g. in trial-wise paradigms or by interrupting continuous presentation at a given moment to obtain confidence ratings). Would the authors agree that, to validate the circular inference model, future studies could test for differences in confidence between perceptual outcomes when there is an asymmetry/bias? If so, I would recommend mentioning this in the discussion.
