# Supplementary material for: A functional theory of bistable perception based on dynamical circular inference
Source: PLoS Comput Biol. 2020 Dec 14;16(12):e1008480. doi: 10.1371/journal.pcbi.1008480 (PMC7769606; doi:10.1371/journal.pcbi.1008480)
Supplement: Attachment — Submitted filename: Response to reviewers.docx [file pcbi.1008480.s006.docx]

**Response to Reviewers**

We would like to thank the editors and reviewers for their thoughtful comments and efforts towards improving our manuscript. Please find below a detailed response to the reviewers’ comments.

**Reviewer #1:**

The authors present a new computational approach toward bistable perception. Based on the established algorithm of circular inference, they deduce minimal conditions under which bistable perception can occur in this framework. With this, the authors place bistable perception in the general context of perceptual inference. Lastly, they relate their model to alternations in perceptual inference related with psychotic symptoms.
Next to analytical methods, the authors performed simulation analyses and compared the model predictions to a number of empirical characteristics of bistable perception (Levelt’s laws and the stabilization of bistable perception by intermittent presentation of ambiguous stimuli).
I think that the circular-inference approach to bistable perception outlined in the manuscript is highly relevant for two reasons: Firstly, it deduces the perceptual dynamics of bistable perception from general considerations of perceptual inference. To my mind, such a functional take on bistability is important, since it may help translate perceptual phenomena observed largely in a laboratory context (eg, the frequent perceptual transitions experienced during perceptions) to the characteristics of perception in real-world scenarios. Secondly, by inverting the circular inference model based on behavior, researchers may be able to quantify the relative contribution sensory evidence and prior knowledge on perceptual inference. This may proffer new opportunities in the study of alterations in perceptual inference (eg, positive psychotic symptoms in schizophrenia).
Although I am very sympathetic toward this work, I have several concerns and wishes for clarification:

General Comments:

**C1:** First, broadly, the authors’ claim that this model can be fitted to behavioral data (and thus be useful to study perceptual function in health and disease) does not seem to be backed up empirically in the manuscript. Personally, I would think that the authors could strengthen their circular-inference model of bistable perception significantly if they could show that the latent variables of the model (eg, weights for ascending/descending loops, feed-forward weight ws, the response variable theta, rates of change ron/roff) can be reliably estimated from data. To my mind, this would mean that, when simulating behavioral responses for a set of latent variables, such latent variables could be recovered from the simulated data.

**R1:** We would like to thank the reviewer for this excellent suggestion. We have now included a “**Parameter Recovery**” section in the **Supplementary Material.**

Second, I have some specific wishes for further clarification regarding the methods and analysis, which I outline in detail below. Lastly, I have a few recommendation on how to improve the connection of the authors’ findings to the existing literature. Specific comments are below.

Major comments:
**C2:** I did not fully understand the role of the decision threshold theta. To my understanding, in this circular-inference (CI) model, setting theta to a sufficiently high value should be necessary to maintain stable perceptual states (view from above; view from below in the example of the Necker Cube). For low values of theta, I could imagine that spontaneous fluctuation in L should lead to frequent switches between dominant perceptual states. Yet, in the method section, the authors note that:
“(…) in the case of continuous presentation, it is necessary to set to a sufficiently high positive value to obtain robust perceptual switches. If = 0, the percept would switch multiple times (just because of the noisy input causing small random fluctuations in ) around the time of perceptual transitions.”

Specifically, I did not understand why the stabilizing effect of a high decision threshold stabilized perception only around the time of perceptual transitions,

**R2:** We thank the reviewer for their comments / questions about theta. They have casted some doubt on the importance of the theta decision criterion and after some reflection, we decided to remove it from the paper and replace it with a simple “maximum-a-posteriori” (theta = 0). The reasons are threefold: First, it’s an ad-hoc addition to the dynamical Circular Inference (dCI) model described in the manuscript (i.e. it is not derived from first principles) and as such, it might be obscuring the take-home message of the paper; second, even in the presence of a non-zero theta, gamma-like histograms (the main reason for adding theta) are generated only in a subset of the parameter space (e.g. for large theta, small sensory gain and mild loops); third, it’s a decision criterion rarely described in the literature. As a result, our dCI model cannot (in its current form) give rise to gamma-like phase duration histograms. We now acknowledge that in the **Main Text (Discussion)** and describe possible solutions, including the filtering of the sensory input, an adaptation-like mechanism (time-dependent transition rates) and the theta decision criterion (we specify that all of them constitute ad-hoc extensions of the model).

Despite the removal of the theta parameter, we would like to briefly address the reviewer’s comment. As they correctly pointed out, the non-zero decision threshold theta introduces a stabilizing factor to the perceptual system, by increasing its robustness against noise. When theta = 0 (MAP decision criterion), a switch occurs each time L (log-odds) crosses chance level (L = theta = 0). The closer L gets to 0, the higher the switching probability, since inputs can push L to the other side of the threshold more easily. When L is in the vicinity of the threshold, tiny fluctuations of the input can cause multiple switches in a very short period of time (“the percept would switch multiple times around the time of perceptual transitions”), resulting in a large number of extremely short (and meaningless) phases (and exponential distribution of phase durations).

When we add a non-zero decision threshold (theta=a), switches occur as follows: when L=a is crossed from below or when L=-a is crossed from above. As a result of this belief-dependent decision threshold, the perceptual system becomes more conservative with regard to switches: a switch occurs only when there is substantial evidence against the current hypothesis; e.g., if the dominant percept switches from SFB to SFA (because L crossed the upper threshold a), it cannot switch back to SFB simply by crossing the same threshold in the opposite direction. Instead, the perceptual system must accumulate evidence in favor of SFB and reach the lower threshold. Therefore, short phases become rare and, under certain circumstances, this can also result in gamma distributions of phase durations.

**C3**: On a more general view, I would find it helpful to see an illustration of the effects of theta on the model’s predictions.

“In our model, a nonzero decision threshold precludes percepts with very short durations. As a result, the distribution resembles a gamma or log-normal distribution. The rising phase and peak of this distribution depend on the decision threshold, but the tail of the distribution does not and is imposed by the mathematical properties of bistable dynamics driven by noise.”

Did I understand correctly that, in the presence of descending loops, it is only due to the decision threshold that simulated phase durations are distributed in a gamma/log-normal distribution? Is the location of the peak of the distribution uniquely determined by the value of the decision threshold? Is there any relation between the energy landscapes shown in Figure 3 and the theta parameter? Moreover, is the minimum value of the decision threshold that is necessary to induce stable perceptual inference correlated with the standard deviation of sensory evidence / the likelihood function?

**R3:** As mentioned above, theta parameter has been removed from the paper, yet we ‘d like to briefly answer the reviewer’s questions.

**“Did I understand correctly that, in the presence of descending loops, it is only due to the decision threshold that simulated phase durations are distributed in a gamma/log-normal distribution?”**

That’s correct. As described elsewhere (see for example Moreno-Bote et al, *J Neurophysiol*, 2007 or more technical textbooks describing the “first-passage time” of bistable attractors), noise-driven attractor models, like the one presented here, generate exponential distributions of phase durations (the peak is at the timescale of the noise). In order to overcome this shortcoming, various researchers (Moreno-Bote et al, *J Neurophysiol*, 2007 etc.) introduced some slow negative feedback (e.g. weak adaptation), which prevents the system from switching rapidly. Such an adaptation-like mechanism can also be included in the dCI framework (e.g. in the form of time-dependent alternation rates $r_{on}(t)$ and $r_{off}(t)$), but results in an overly complex model which could obscure the take-home message of the manuscript (which is the potential role of loops in the generation and dynamics of (bistable) perception). We hope to present this extended dCI model in a future publication.

**“Is the location of the peak of the distribution uniquely determined by the value of the decision threshold?”**

The peak depends on theta and on the dynamics (i.e. descending loops, alternation rates and sensory gain)

**“Is there any relation between the energy landscapes shown in Figure 3 and the theta parameter?”**

None. All Bayesian models consist of 2 parts: an inference model (posterior estimation and belief updating) and a response model (transformation of posteriors into (perceptual) decisions). Eq (1) and (6) (they are visualized in Fig. 3) formalize the former, while theta parameterizes the latter. Note that in Figure 3 we make no reference to percepts, only to beliefs (L), and theta (e.g. thata=$\pm$a) could be illustrated as two vertical lines: L=a and L=-a (when the dot crosses L=a from right to left, the dominant percept switches from SFA to SFB; the opposite happens when the dot crosses L=-a from left to right).

**“Moreover, is the minimum value of the decision threshold that is necessary to induce stable perceptual inference correlated with the standard deviation of sensory evidence / the likelihood function?”**

The minimum value depends on the variance of the noise, but the relationship is non-linear, since it also depends on loops, switch rates etc. We would like to clarify that, in most cases, the phase duration histogram is either very positively skewed (exponential) or very negatively skewed (complete stability), and it gets a gamma-like shape for a narrow range of parameter values.

**C4**: In a related point, I would need additional clarifications on how the role of theta relates to the function of descending loops:
“Note that large values of can lead to a distribution of phase duration similar to the system of descending loops. However, while the distribution of phase duration cannot be considered proof of the presence of circular inference, the resulting confidence is often below the decision thresholds. This may preclude the emergence of strong and stable percepts in the absence of descending loops.”

Here, the authors introduce the concept of “confidence”. If I understood it correctly, high-confidence perceptual states only emerge in the presence of descending loops. I would find it helpful if the authors could contextualize this to the existing literature on bistable perception:
A number of studies has devoted a lot of attention of mixed percepts during bistability (eg., Knapen 2011). Do the authors assume that such mixed percepts (low-confidence/high-uncertainty perceptual states) arise at the time of state transitions between the energy wells in Figure 3c? How would the energy landscape look like for other types of bistable stimuli that show sudden transitions, such as discontinuous structure-from-motion stimuli (eg., Weilnhammer 2013)?

**R4: ‘In a related point, I would need additional clarifications on how the role of theta relates to the function of descending loops’**

We would like to clarify that descending loops, but not theta, change the dynamics of the system (from leaky integration to bistable dynamics). Descending loops have a profound effect on belief updating, while theta describes how the posteriors are transformed into perceptual decisions.

**‘Here, the authors introduce the concept of “confidence”. If I understood it correctly, high-confidence perceptual states only emerge in the presence of descending loops.’**

In this paper, the terms “confidence” and “posterior probability” (log-odds) are used interchangeably; they both refer to variable *L* (note that both $X_{2}$and $X_{3}$ are binary variables). As correctly pointed out by the reviewer, high-confidence perceptual states (together with high stability) only emerge in the presence of descending loops. This is depicted (for instance) in **Fig. 3c** and **4a.** Stronger loops result in more positive (and negative) stable fixed points (belief / confidence hovers around those attractors).

**‘A number of studies has devoted a lot of attention of mixed percepts during bistability (eg., Knapen 2011). Do the authors assume that such mixed percepts (low-confidence/high-uncertainty perceptual states) arise at the time of state transitions between the energy wells in Figure 3c?’**

This is an interesting speculation. It is likely that when the perceptual system holds a weak belief ($L\approx0$), none of the 2 interpretations can clearly dominate, giving rise to mixed percepts. This seems compatible with the findings in (Knapen et al, 2011): rFPC might indicate that the belief is near the decision threshold ($-\varepsilon<L<\varepsilon$), hence a perceptual switch is probable. This possibility is now discussed in the **Main Text (line 327):**

**“**instead, low confidence might give rise to mixed percepts (Knapen et al, 2011)**”**

**‘How would the energy landscape look like for other types of bistable stimuli that show sudden transitions, such as discontinuous structure-from-motion stimuli (eg., Weilnhammer 2013)?’**

If a similar generative model can describe the “structure-from-motion” stimulus (this seems plausible; see **Fig 1**), then the energy landscape (dynamics) would be the same. Instead, the difference with the Necker Cube might lie within the noise distribution (sensory input). For example, in the case of the Lissajous figure, the shape of the stimulus is changing; thus the illusory depth cues (and consequently $\mu_{noise}$) are frame-dependent and there is higher switching probability when ambiguity is higher. Alternatively, the decision criterion might be task- (and stimulus-) dependent (e.g. in the case of theta decision criterion, parameter theta might depend on the task – smaller theta could result in more abrupt switches if mixed percepts arise when the belief lies between the two thresholds).

**C5**: With regard to perceptual biases: From Figure 3d, should it be concluded that the CI model assumes a difference in confidence when there is a bias between perceptual alternatives? In the example of the Necker Cube, this would mean that the view-from-below is generally associated with reduced confidence. To my mind, this would be an important prediction of the CI model. Are the authors aware of any empirical evidence for this model prediction?

**R5:** We would like to thank the reviewer for this excellent suggestion. This is indeed a strong prediction of the dCI model: When there is an asymmetry, the weaker interpretation is associated with reduced confidence. That’s because a bias affects the depth and the position of the wells (**Fig. 3d**). That being said, testing this prediction could be problematic for two reasons. First, because it is very hard to (reliably) measure confidence in a bistable perception experiment, without interfering with bistability. Second, because the magnitude of the effect might be small, especially compared to the effect of bias on stability (see **Fig 2c**). Consequently, we are not aware of any evidence supporting (rejecting) this prediction.

**C6**: In the section on Levelt’s 4th law, the authors investigate the effect on an increase in the strength of both interpretations on the alternation rate of a bistable stimulus. They captured this increase in stimulus strength by increasing the variance of the noise distribution. This choice did not seem straightforward to me. Several alternatives would also seem plausible to me: Could an increase in stimulus strength be reflected by a decrease in variance of the noise distribution? Or by a modulation in variance of the likelihood distribution?

**R6**: In this paper we modelled sensory evidence as a Gaussian process. At each time step, a sample is drawn from a Gaussian distribution; the more positive (negative) the sample, the stronger the (new) evidence for SFA (SFB). By increasing the variance of this Gaussian (noise) distribution, we increased the dispersion of the samples around 0 (in the ambiguous case). In other words, the mean of the absolute value of the inputs was larger, while the mean of the input remained the same. That’s crucial, because Levelt’s 4^th^ law (unlike laws 1-3) describes what should happen when we increase the strength of both interpretations equally.

**‘Could an increase in stimulus strength be reflected by a decrease in variance of the noise distribution?’**

Not really. A decrease in the variance of the noise would result in weaker inputs (the mean of the absolute value of the inputs would be smaller, see **Figure below**).

**‘Or by a modulation in variance of the likelihood distribution?’**

This wouldn’t work either; the stimulus strength is reflected, first and foremost, in the world-external model (noise distribution) and not in the internal model (likelihood function).

Minor comments

With regard to the Abstract, I would like to make a few suggestions that could render the content more accessible to the naïve reader:

**C7**: While these points become clear after reading the manuscript, my personal impression was that they are difficult to understand on the basis of the abstract and general knowledge about bistable perception. Readers without a background in computational modelling of bistable perception might have a hard time understanding these points.

“We show that in the face of ambiguous sensory stimuli, circular inference can
turn what should be a leaky integrator into a bistable attractor switching between two highly trusted interpretations. (…) Since it is related to the generic perceptual inference mechanism, this approach can be used to predict the tendency of individuals to form aberrant beliefs
from their bistable perception behavior.”

**R7:** We agree with the reviewer that the abstract should be accessible to as many researchers, as possible, including people without a theoretical background. Unfortunately, since this is a theoretical paper, it was not possible to avoid all technical terms. Nonetheless, we rewrote those parts of the abstract as follows:

“We show that in the face of ambiguous sensory stimuli, circular inference can change the dynamics of the perceptual system and turn what should be an integrator of inputs into a bistable attractor switching between two highly trusted interpretations. (…) Since it is related to the generic perceptual inference and belief updating mechanisms, this approach can be used to predict the tendency of individuals to form aberrant beliefs from their bistable perception behavior.”

**C8**: Maybe I have overlooked something, but while the main text contains a section of psychotic symptoms in schizophrenia patients, I could not find a discussion on cognitive functions in non-clinical populations.

“Overall, we suggest that feedforward/feedback information loops in hierarchical neural networks, a phenomenon that could lead psychotic symptoms when overly strong, could also underlie cognitive functions in nonclinical populations.”

**R8:** We have replaced “cognitive functions” with “perception”.

With regard to the introduction, I have a few additional comments:

**C9**: If I understood correctly, the authors introduced perceptual inference during bistable perception as “suboptimal”:

“In most cases, this task is performed very accurately, and the correct interpretation is found. Sometimes, perceptual systems fail to detect any meaningful interpretation (e.g., when sensory evidence is too degraded) or converge to the wrong interpretation. Finally, a third possibility occurs (mainly in lab conditions [3]) when ambiguity is high; the system detects more than one plausible interpretations but instead of committing to one interpretation, it switches every few seconds, a phenomenon known as bistable perception [4].

(….) Crucially, there is a discrepancy between the real input and the input assumed by the internal model. This, together with the loops, predicts the suboptimal inference at the heart of bistable perception (Figure 1; caption).

I was wondering whether the authors could add a little more detail as to why they view perceptual inference is suboptimal or incorrect. As they authors note throughout the manuscript, truly ambiguous images (eg. the line drawings of a Necker cube, disparate monocular inputs in case of binocular rivalry) are very rare. Could it also be that, because of the extremely low probability of a fully ambiguous real-word cause of sensory input, committing to one highly trusted stimulus interpretation is indeed adaptive/optimal? This thought also appears in the discussion (“Moderate descending loops could improve the system, allowing rapid and robust decisions even when evidence is not conclusive”)

**R9:** In the paper we put forward an interpretation of the phenomenon of bistable perception that relies on two premises: 1) there is a discrepancy between objective reality and the internal representation learnt by the brain and 2) perceptual inferences are “suboptimal”, due to the presence of information loops.

The first premise is straightforward (see **Methods**): when we look at the Necker Cube, we see a 2D image that contains illusory depth cues. Our brain interprets those cues as an indication of “real depth” and generates a 3D image that is compatible with the 2D input. We highlight that this does entail suboptimal inferences, since optimality can be assessed only with regard to the internal model.

The second premise is less straightforward. In the **Results** section, we show that a Bayesian observer that does sensory integration cannot explain the phenomenology of bistable perception. A perceptual system that is corrupted by descending loops (prior amplification), by contrast, is compatible, both with the phenomenology and the empirical observations about bistability. We note that circular inference is by definition suboptimal, since it violates Bayes theorem (priors are overcounted).

Suboptimal inferences could, under certain circumstances, be adaptive. For example, we argue in the **Discussion** that loops offer a comparative advantage in cases of high uncertainty, when all possible actions (which depend on perceptual inferences) are better than “no action”.

**Reviewer #2:**

General comment
In this paper, Pantelis Leptourgos and colleagues propose 'circular inference', a model of hierarchical Bayesian inference, for the modeling of bistable perception. They suggest that bistability can be conceived as a process whereby sensory responses trigger activity in higher-level areas but are also modulated by feedback projections from these same areas, thus reverberating prior beliefs in the cortical hierarchy. They show that in the face of ambiguous sensory stimuli, circular inference can turn what should be a leaky integrator into a bistable attractor that switches between two alternative interpretations. Moreover, they report that their model is able to capture various aspects of bistable perception, including Levelt’s laws and the stabilizing effect of intermittent stimulus presentation. They speculate that feedforward/feedback information loops in hierarchical neural networks, a phenomenon that could lead to psychotic symptoms when overly strong, could also underlie cognitive functions in nonclinical populations.
Overall, this is a very interesting, timely and well-written paper that adds an interesting new approach to the modeling of bistable perception. Since this algorithmic model is also based on established theories of cortical neural circuit function, such as E/I balance it is a promising candidate to link modeling at the algorithm level to the level of neural implementation. In addition, it may be well suited to the modeling of altered perceptual inference in neuropsychiatric disorders such as schizophrenia, as the authors point out. I have a few concerns and questions that I would like to ask the authors to consider. In particular, there seem to be a few problems with the part regarding Levelt's laws that need to be addressed (see below for details).

Specific points

**C1**: In the introduction, the authors state that a few important questions have remained unanswered by previous modeling approaches. The first is, why a system should form "such strong percepts based on unreliable sensory evidence". I'm not sure that the sensory evidence that typically gives rise to bistable perception is what I would call "unreliable". It is perceptually ambiguous, which means that it is compatible with two competing perceptual interpretations. However, as opposed to noisy stimuli, the evidence is quite clear-cut and reliable. It's not the sensory information that's unreliable, it's the perceptual system that has a hard time "making up it's mind" which of two clear-cut interpretations to go for. It is therefore not obvious to me what should be puzzling about the fact that two "strong" percepts are formed.

**R1:** We would like to thank the reviewer for this excellent remark. We agree that it might be misleading to call the bistable stimulus “unreliable”, so we replaced it with the term “ambiguous”. The point raised here is the following: The cues are strong and reliable (when used separately; e.g. in the case of a disambiguated Necker Cube), but they are also contradictory. When taken together, the overall evidence for any interpretation is weak (In reality, the following is true for the likelihood function, in the ambiguous case: E(P(S|X=SFA)) ~ E(P(S|X=SFB)) ~ 0.5).

In our view, the hardest obstacle for any normative account of bistability is the combination of high confidence (question 1) and persistence (question 2 – see below), so we now present them as 1 outstanding question.

The second question is why percepts persist "instead of switching rapidly". To my mind, this question has been answered quite satisfactorily, from a normative perspective, by Hohwy et al. 2008. In their conceptual model (which was later implemented as a computational model, see Weilnhammer et al. 2017), it is the current percept that determines the prediction of what the sensory input is caused by. This prediction is fed back from higher to lower hierarchical levels and thereby stabilizes perception. This is very similar to what the authors suggest in their current work, so I'm struggling to see what is new here (again, from a normative perspective).

(Hohwy et al, 2008) and especially (Weilnhammer et al, 2017) both described a very interesting model of bistable perception that constitutes an important contribution to the field for various reasons: 1) it is inspired by the influential predictive coding framework 2) it can be fitted to data - just to name a few. That said, it is not clear to us whether this is a purely normative model, or a descriptive model that shares certain features with established Bayesian models. More particularly:

- The model that is described in (Hohwy et al, 2008) and formalized in (Weilnhammer et al, 2017) is largely based on a simplified predictive coding model, often called Kalman filter (a linear quadratic estimator). Crucially, inferences made by such a scheme are optimal only under certain assumptions, in particular when the variables are Gaussian and decisions are based on a “maximum-a-posteriori” decision criterion. In the context of bistable perception (high level (perceptual) decision making more generally speaking), it is unclear whether Gaussian (continuous) variables are meaningful or whether they should be replaced by binary (discrete) variables, in which case Kalman filters like the one described in the aforementioned papers are inappropriate (alternatives exist: e.g. Hierarchical Gaussian Filter (Mathys et al, 2011;2014); Variational message passing algorithms and Belief Propagation (Friston et al, 2017); but all of them are high-complexity models that rely on slightly different assumptions).
- Even if Gaussian variables are applicable here, it is not clear whether the suggested belief updating scheme is doing optimal inference. Bayesian belief updating is largely based on the following idea; the posteriors at time (t) constitute the priors at time (t+1) (one can add dynamics, as in the dCI model), which are compared to sensory inputs. The residual (prediction error) is used to update the prior, which gives rise to the posterior at time (t+1). This simple optimal scheme will very quickly converge to the correct posterior, that is a symmetrical bimodal distribution where interpretations are equally strong. Instead, (Weilnhammer et al, 2017) postulate an additional stability prior that depends on the current interpretation. In our view, a justification of such a prior from a normative point of view is problematic - we would like to highlight that the simple belief updating scheme presented above also contains implicitly a “stabilization prior”, which depends on the belied and not on the percept.
- Additionally, there are several ad-hoc assumptions which cannot be derived from first principles. For example, there is an exponential decay mechanism (π_stab(t+1) = π_stab(t)*exp(-|PE(t)|)) that destabilizes the current percept. Importantly, the value of π_stab is renormalized after each switch (π_stab=π_init). It’s thanks to this renormalization that the model predicts strong and stable percepts, yet this mechanism is poorly explained from a normative perspective.

The above scrutiny should not be interpreted as a doubt about the high importance of the model presented in (Hohwy et al, 2008) and (Weilnhammer et al, 2017). Instead, it is, in our humble opinion, a proof that there is still a lot to be done on the computational underpinnings of bistable perception, especially from a normative perspective.

The third question is "how the behavior of individuals in bistable perception tasks may predict their performance in other probabilistic inference tasks". This is a potentially interesting topic, but I did not find it addressed in this paper.

We believe that this is only partly true. It is true that in this manuscript we present a computational model of bistable perception. Yet, the model is based on a generic mechanism of probabilistic inference, which has been used in the past to explain the behavior of participants in different tasks (a probabilistic reasoning task (Jardri et al, 2017)). We think that it might be possible to predict the behavior of participants in bistable perception tasks by looking at the recovered parameters from different tasks, e.g. from probabilistic reasoning data (and vice versa) – participants who overcount their priors in a probabilistic reasoning task (perhaps due to descending loops) could exhibit higher stability and confidence. We might explore this prediction in future work.

**C2**: Related to the second of the above questions: From a normative perspective and conceptually, the circular inference model of bistable perception is extremely similar to the predictive coding model proposed by Hohwy et al./Weilnhammer et al.. Both models assume top-down projections (feedback/descending loops) to stabilize and bottom-up projections (feedforward/ascending loops) to destabilize perception. Since these two models are so closely related, I think it would be worthwhile to add a paragraph to the discussion section where the circular inference model is discussed in the light of this previous model, how the new model differs from the predictive coding model, and how it may go beyond it?

**R2:** We thank the reviewer for this excellent suggestion. We have now added a paragraph in the **Discussion** in which we compare the predictive coding model described in (Weilnhammer et al, 2017) with the dynamical circular inference model presented here.

**“**In particular, an interesting model that bears some similarity with the dCI model was described by Hohwy and Friston [17] and formalized by Weilnhammer and colleagues [18]. Like dCI, it relies on a message passing algorithm, but instead of belief propagation, it is largely based on a simplified version of predictive coding [28,66,67] - predictive coding postulates that priors explain away sensory inputs while residual prediction error signals are fed-forward to higher regions to update beliefs. Importantly, top-down effects play a crucial role in both explanations of bistability. Instead of adding (descending) loops, the predictive coding model suggests that perception is biased by a stabilization prior, which depends on the current interpretation. This prior is constantly weakened by prediction errors emerging from evidence for the suppressed percept, via an exponential decay mechanism. A switch occurs when the evidence for the suppressed percept surpasses that for the dominant percept. Despite their similarities, the two models are not identical. While dCI is derived from first principles (inference in a hidden markov model, corrupted by loops), the predictive coding model relies on a number of ad-hoc assumptions, that nuance its normative character. For example, the precision of the stabilization prior is renormalized after each switch, resulting in strong and stable percepts; this is an important assumption, yet it’s difficult to interpret it from a normative perspective.**”**

**C3**: Why is the theta parameter needed at all, as it only has a thresholding function to avoid fast reversals. It is not needed in the presence of descending loops, and is stated therefore not to be considered further (Lines 352 f.)? So why is it needed in the model at all? And what could be an equivalent of this parameter in terms of a neural mechanism or function? Please clarify.

**R3:** As we explained above (see C2 – C4, Rev 1), we finally decided to remove parameter theta from the manuscript and replace the “theta” decision criterion with a simple “maximum-a-posteriori”. The shortcoming of the simplified model is that it cannot generate gamma-like distributions of phase durations. We now suggest various solutions in the **Discussion**.

**C4**: Maybe I din't get the point here, but I'm not sure I understood why sensory evidence is referred to as "noise" (e.g. line 418). The sensory data do contain some information, not only noise, don't they?

**R4:** It is true that sensory data contain useful information. Yet, the term “noise” (or “noise distribution”) is very common both in the field of Bayesian modelling (it refers to the distribution of the measurement s for a given stimulus x; see for example (Ma, Kording and Goldreich, 2012)) and in the field of dynamical systems (it refers to the perturbation that pushes the dynamical variable L away from the stable fixed point).

**C5**: Levelt's 2nd law is misrepresented and it therefore seems that the authors' conclusions in this regard are not tenable. It is stated that manipulating the strength of one perceptual interpretation "mainly affects the persistence of the stronger interpretation". This is not what Levelt's 2nd law says. In fact it states that increasing stimulus strength for one eye will NOT affect the average perceptual dominance duration of that eye’s stimulus. Instead, it will reduce the average perceptual dominance duration of the other eye’s stimulus. What results from the model presented here is exactly the opposite.

**R5:** We thank the reviewer for this comment. They are right that the original formulation of Levelt’s 2^nd^ law is the following (it referred to binocular rivalry): “Increase in stimulus strength in one eye will not affect mean dominance duration for the same eye” (Levelt, 1966). But more recently, it was found that the original propositions are not completely compatible with the empirical evidence (Brascamp et al, 2006; Kang, 2009; Moreno-Bote et al, 2010; Platonov and Goosens, 2013; etc.). With regard to Levelt’s 2^nd^ law, those studies showed that when one eye’s strength is fixed to a relatively low level while the other eye’s strength was varied, the largest change in dominance durations occurred for the variable-strength eye, in disagreement with Levelt’s 2^nd^ law’s original prediction. In a paper published on the 50^th^ anniversary of Levelt’s original monograph, Jan Brascamp and Christiaan Klink (Levelt also made a contribution) suggested a set of four modified laws that encapsulated the progress that had been made since the 60s ((Brascamp et al, 2005); see also attached Figure (Fig 3 of the aforementioned paper)). The 2^nd^ law was modified as follows: “Increasing the difference in stimulus strength between the two eyes will primarily act to increase the average perceptual dominance duration of the stronger stimulus.”. In 2008, Klink and colleagues showed that Levelt’s laws can also be applied to perceptual rivalry (e.g. Necker Cube) and suggested the following formulation for the 2^nd^ law: “Manipulations of stimulus strength of one perceptual interpretation of a bistable stimulus will mainly influence the average dominance duration of the perceptual interpretation corresponding to the strongest stimulus” (Klink et al, 2008). In this manuscript we used Brascamp’s / Klink’s formulation, which can be applied to the Necker Cube.

***(Brascamp et al, 2005)***

We now insist more on the fact that we are using the new formulations of the laws (**Lines 410 - 411**).

**C6**: Levelt's 3rd law is similarly misrepresented. The authors state "… that increasing the difference in the stimulus strength between the two perceptual interpretations should result in a decrease in the perceptual alternation rate". Again this is not what Levelt's 3rd says. What it does state is that increasing the strength of one stimulus will increase the alternation rate. Increasing the strength of one stimulus effectively increases the difference in stimulus strength, therefore the authors' statement says exactly the opposite and, again, what results from the model does not conform to Levelt's 3rd law.

**R6:** See also response to C5. The original formulation (Levelt, 1966) of Levelt’s 3^rd^ law was the following: “Increase of the stimulus strength in one eye will increase the alternation rate”. In (Brascamp et al, 2005), the 3^rd^ law was reformulated as follows: “Increasing the difference in stimulus strength between the two eyes will reduce the perceptual alternation rate”. Finally, Klink’s formulation (Klink et al, 2008) was the following: “Manipulating the stimulus strength of

one perceptual interpretation of a bistable stimulus will influence the average rivalry reversal rate.”

**C7**: The model manipulation that is used to test Levelt's 4th law is inconsistent with what was done to test the other three laws. For the first three laws, which entail a difference in stimulus strength, stimulus differences are modeled by changing the noise drift parameter. This is obviously not possible if the strength of both stimuli needs to be changed in the same direction. Instead, the authors resort to another parameter, the noise distribution. In other words, this looks like the authors model changes in stimulus strength by changing ad libitum whichever model parameter suits them best. There may be a good reason to do it this way, but if there is, it should be explained.

**R7:** See also our response to C6, Rev1. We believe that it is not fair to say that we manipulated parameters ad libitum. Since this is a normative (bayesian) model, each parameter has a very specific meaning – all parameters are related either to the task (external / world model – e.g. sufficient statistics of the noise distribution ($\mu_{noise},\sigma_{noise}$)) or to the internal processing of the sensory input (internal model – e.g. sufficient statistics of the likelihood function ($\mu_{int},\sigma_{int}$)). As a result, the choice of the parameter to be manipulated is dictated by its interpretation. For example, a change in the stimulus strength corresponds to manipulations of the external model. For laws 1-3, in which we change the strength of one interpretation only, our manipulation should result in biased measurements – i.e. we should change the mean of the noise distribution. On the other hand, law 4 describes what happens when we change the strength of both interpretations equally. That speaks to an increase in the mean of the absolute value of the measurements, while the average measurement remains unchanged – the easiest way to achieve that is through an increase in the variance of the noise. This is now clarified in the text (**Lines 464 - 466**)

C8: Line 324: "resulting in an exponential decay observed in the distribution of dominance durations". It would be very helpful if the dominance distributions were presented in a figure.

**R8:** The exponential phase duration histogram is now presented in the **Supplementary Material, Figure S7.**

**C9**: Line 127: It is misleading to say that the Necker cube is "equally compatible with 2 different 3D cubes", as it is actually more compatible with the view from above (as the authors also state further down in the paper).

**R9:** We removed the word “equally”

**C10**: Line 348: "see the Results section". This IS the results section.

**R10:** We removed the phrase

**C11**: "Severity of loops" (used multiple times) should probably better read "Strength of loops".

**R11:** We replaced “severity” with “strength”

**C12**: Line 714: "flying" should read "fleeing".

**R12:** We replaced “flying” with “fleeing”

Signed: Philipp Sterzer
